# Development of the arcuate fasciculus is linked to learning gains in reading

**DOI:** 10.1162/imag_a_00542

**Published:** 2025-04-17

**Authors:** Ethan Roy, Emily M. Harriott, Tin Q. Nguyen, Adam Richie-Halford, Ariel Rokem, Laurie E. Cutting, Jason D. Yeatman

**Affiliations:** Graduate School of Education, Stanford University, Stanford, CA, United States; Vanderbilt Brain Institute, Vanderbilt University, Nashville, TN, United States; Peabody College of Education and Human Development, Vanderbilt University, Nashville, TN, Unite States; Vanderbilt University Institute of Imaging Science, Vanderbilt University, Nashville, TN, United States; Division of Developmental-Behavioral Pediatrics, Stanford University, Stanford, CA, United States; Department of Psychology and eScience Institute, University of Washington, Seattle, WA, United States

**Keywords:** white matter, reading, mathematics, diffusion MRI, longitudinal analysis

## Abstract

Past studies leveraging cross-sectional data have raised questions surrounding the relationship between diffusion properties of the white matter and academic skills. Some studies have suggested that white matter properties serve as static predictors of academic skills, whereas other studies have observed no such relationship. However, longitudinal studies have suggested that within-individual changes in the white matter are linked to learning gains over time. In the present study, we look to replicate and extend the previous longitudinal results linking longitudinal changes in the white matter properties of the left arcuate fasciculus to individual differences in reading development. To do so, we analyzed diffusion MRI data, along with reading and mathematics scores in a longitudinal sample of 340 students as they progressed from first grade into fourth grade. Longitudinal growth models revealed that year-to-year within-individual changes in reading scores, but not mathematics, were related to the development of the left arcuate fasciculus. These findings provide further evidence linking the dynamics of white matter development and learning in a unique sample and highlight the importance of longitudinal designs.

## 1 Introduction

Over the course of the lifespan, an individual’s experiences shape the development of white matter tracts, the large bundles of axons connecting distinct cortical areas. Experience has been shown to sculpt the properties of these white matter tracts on the time scale of hours (Sagi et al., 2012) to weeks and months (Huber et al., 2018). The acquisition of academic skills that rely on culturally invented symbolic systems serves as a prominent example of experience-dependent white matter plasticity. Although the human brain has not evolved to innately process and manipulate symbolic representations of language and number, its incredible capacity for plasticity allows it to adapt in response to repeated exposure to these cultural inventions to give rise to complex behaviors, such as reading and mathematics (Caffarra et al., 2021;Dehaene, 2005;Dehaene & Cohen, 2007).

Over the past few decades, advances in diffusion MRI have allowed researchers to study human white matter*in vivo*through various metrics calculated using the diffusion MRI signal. Diffusion metrics, such as fractional anisotropy (FA) and mean diffusivity (MD), describe how water molecules diffuse through brain tissue and serve as indirect measures of the underlying neural tissue. Studies using animal models have demonstrated a relationship between differences in diffusion metrics and changes in the underlying white matter, such as demyelination in response to neuronal injury or illness (Song et al., 2002,^2003^,^2005^). Furthermore, diffusion metrics have been shown to align with histological measures of myelin in post-mortem samples of white matter tissue (Seehaus et al., 2015). However, it bears mentioning that metrics derived from diffusion MRI measurements remain indirect measures and do not necessarily provide direct insight into the properties of the underlying tissue (Budde et al., 2007;J. Zhang et al., 2012). Nevertheless, diffusion MRI has proven to be a critical tool in furthering our understanding of human white matter.

Past research leveraging diffusion MRI has led to mixed, and sometimes conflicting, conclusions about the relationship between white matter properties and academic skills, such as reading and mathematics. In the case of reading, some cross-sectional studies have suggested that higher fractional anisotropy (FA) in the left arcuate fasciculus, inferior longitudinal fasciculus (ILF), and superior longitudinal fasciculus (SLF) correlates with higher reading scores (Welcome & Joanisse, 2014) and that individuals diagnosed with dyslexia demonstrate lower FA in these same pathways (Van Der Auwera et al., 2021;Vanderauwera et al., 2017;Welcome & Joanisse, 2014). However, other studies have observed the opposite relationship, with higher levels of reading skill corresponding to lower levels of FA in these tracts (Yeatman et al., 2011) or no relationship whatsoever between reading skill (or dyslexia) and white matter properties (Meisler & Gabrieli, 2022;Moreau et al., 2018;Roy, Richie-Halford, et al., 2024).

These mixed findings suggest that past results reporting a cross-sectional relationship between academic skills and white matter may stem from idiosyncratic properties of the study sample. In fact, past studies incorporating measures of socioeconomic status into their analyses have demonstrated that the relationship between reading skill and white matter properties varies as a function of socioeconomic status (Gullick et al., 2016;Ozernov-Palchik et al., 2019;Turesky et al., 2022). Based on these findings, the reported link between reading skill and white matter properties may stem from characteristics of the sample populations, such as environmental, demographic, or socioeconomic factors, and may not generalize to the broader population.

In the case of mathematics skills, FA in the left SLF, left ILF, and left arcuate has been shown to predict arithmetic ability (Jolles et al., 2016;Matejko & Ansari, 2015;Matejko et al., 2013;Tsang et al., 2009;Van Beek et al., 2014;van Eimeren et al., 2008). In addition to this set of left lateralized tracts, studies have also found that the diffusion properties of right hemisphere tracts also correlate with mathematics abilities (Cantlon et al., 2009;Polspoel et al., 2019;Rykhlevskaia et al., 2009;Till et al., 2011), suggesting that the neural circuitry underlying mathematics skill might involve a bilateral collection of white matter tracts. These findings again suggest that individual differences in white matter properties might relate to differences in mathematics performance. However, as with past studies of reading, these findings are based on small scale, cross-sectional, samples of convenience and have yet to be replicated using large, representative, multi-site consortium data sets.

In contrast to the mixed findings observed in cross-sectional studies, longitudinal and intervention studies using repeated measures to evaluate within-individual change over time have demonstrated a consistent relationship between changes in the white matter and reading and mathematics development. Observational studies have shown that within-individual gains in reading skill are associated with the development of the left arcuate fasciculus (Myers et al., 2014;Roy, Richie-Halford, et al., 2024;Wang et al., 2017;Yeatman, Dougherty, Ben-Shachar, et al., 2012) and that delayed development of frontal-parietal and posterior parietal white matter networks is associated with difficulties in mathematics (Ranpura et al., 2013). Furthermore, targeted learning intervention studies have demonstrated that intensive educational experiences can drive changes in an individual’s white matter properties that are associated with behavioral gains in reading skills (Huber et al., 2018;Meisler et al., 2023) and in mathematics (Jolles et al., 2016;Klein et al., 2019). Together, these results suggest that properties of the white matter tracts associated with academic skills change over time and, in fact, change in response to the educational opportunities and experiences afforded by an individual’s environment.

A recent study byRoy et al. (2024)aimed to clarify past findings and evaluate both the cross-sectional and longitudinal hypotheses surrounding the relationship between white matter and reading skill. In a sample of children aged 5–10 years, within-individual changes in the diffusion properties of the left arcuate related to within-individual gains in reading skill based on the PLING sample (Wierenga et al., 2018). Interestingly, these analyses did not find any cross-sectional differences between reading skill and white matter properties at a given time point, and three other data sets totaling 14,249 participants confirmed the lack of a generalizable cross-sectional relationship between white matter and reading. Furthermore, models examining the temporal dynamics of reading gains and the development of the left arcuate suggested that improvements in reading skills precede changes in the white matter. Together, these results suggest that the white matter properties of the core reading circuitry change over time, potentially in response to environmental and educational factors that promote the development of reading skills.

In the present study, we look to replicate the findings reported inRoy et al. (2024)using unique diffusion MRI data collected from an independent longitudinal sample of children between 6 and 8 years of age, from a completely different regional context in the United States (the southern United States, whereas the PLING sample was collected in California). We use pyAFQ (Kruper et al., 2021) to examine the development of the left and right arcuate fasciculus and their relationship with the development of reading and mathematics skills. The use of pyAFQ to generate tract profiles allows us to leverage non-linear modeling approaches, such as generalized additive mixed models (GAMMs;Hastie & Tibshirani, 1987), to examine the development of white matter properties over the entire length of each tract, as opposed to the approach used inRoy et al. (2024), which relied on a single average diffusion metric for each tract. This approach to modeling tract profiles is appealing, as diffusion properties vary over the length of a tract and important group differences in these diffusion properties may be obscured by collapsing these data into a single value (Muncy et al., 2022;Yeatman et al., 2012).

Our analyses reveal that, within this longitudinal sample, on average, within-individual changes in the mean diffusivity (MD) of the left arcuate fasciculus, but not the right arcuate, are related to within-individual changes in reading scores. However, in both the left and right arcuate, we did observe an interaction between tract position and within-individual gains in reading. There was no such relationship between development of the left arcuate and mathematics skill.

To explain these findings, we examined between-individual differences in the growth trajectories of both white matter and academic skills and found significant interindividual variation in both reading and white matter development, but not in mathematics skill. Furthermore, modeling the time series of MD development and reading development suggested that individual gains in reading skill precede changes in the white matter properties along the length of the left arcuate fasciculus. These findings serve to replicate past findings demonstrating a longitudinal relationship between reading skill and white matter development and highlight the importance of examining longitudinal development across the length of an entire tract.

## 2 Methods

### 2.1 Participants

The data used in the present study came from a 4-year longitudinal study examining the development of academic behaviors and brain properties between first and fourth grades. In total, 340 first graders were recruited from the greater Nashville area to participate in this longitudinal study of the development of academic skills. Starting in the summer after first grade, participants completed annual behavioral and neuroimaging sessions in which they completed either the Woodcock–Johnson III or IV assessments of basic reading and mathematics (Mather & Jaffe, 2016;R. W. Woodcock et al., 2001), as well as structural and diffusion neuroimaging scans. Behavioral and neuroimaging data were collected across four waves. The first time point involved the collection of neuroimaging and/or behavioral data from 268 participants, at the second time point from 220 participants, at the third time point from 183 participants, and at the fourth time point from 92 participants.

At the onset of the study and at each observation, parents provided informed consent and participating children provided assent before data collection. Data collection was carried out with approval from the Vanderbilt University Institutional Review Board. Data sharing was carried out with approval from Vanderbilt University and Stanford University Offices of Sponsored Research.

Across all participants, 276 individuals completed at least one concurrent neuroimaging and behavioral session. Of these participants, 63 completed just one session, 59 completed two sessions, 97 completed three sessions, and 57 completed all four behavioral and neuroimaging sessions. Because we are interested in examining the within-individual dynamics of white matter, reading, and mathematics development, we limited our analyses to participants who completed three or more sessions, as these dynamic systems cannot be studied with two or fewer observations per participant. Furthermore, there were seven sibling pairs in the data set. To avoid any potential confounds due to family effects, we randomly excluded one participant from each sibling pair.

After controlling for image quality (see*Neuroimaging Data*for overview on quality control procedures), we had a final sample of 101 participants (52 female participants, and 49 male participants) with the necessary behavioral and neuroimaging data at a minimum of three time points. For our longitudinal analyses, we examined Woodcock–Johnson W scores. The Woodcock–Johnson W score is a mathematical transformation of the Rasch Model on an equal-interval scale (Woodcock, 1999;Woodcock & Dahl, 1971), meaning that they are particularly well suited for examining within-individual growth in a given metric.

At the first time point, participants were, on average, 7.45 years old (SD = 0.34). The variation in age was relatively consistent across all four time points, with a minimum standard deviation of 0.339 years and maximum standard deviation of 0.360 years, and displayed an average Woodcock–Johnson W score in Basic Reading of 478.24 (SD = 19.80) and an average Woodcock–Johnson W score in mathematics of 466.26 (SD = 14.24). The Woodcock–Johnson Basic Reading scores used in this analysis were calculated as the average W score on the word identification and word attack sub-tests and the Woodcock–Johnson mathematics scores were calculated as the average W score on the calculations and applied problems sub-tests. Across all observations, 15 participants in the final sample had at least one Woodcock–Johnson standard score of less than 85, a conventional cutoff for receiving a dyslexia diagnosis. Across all four time points, 42 participants did not complete Woodcock–Johnson mathematics assessment, with 37 of these missing observations occurring during the first observation.

### 2.2 Neuroimaging data

At each time point, MRI data were acquired at Vanderbilt University Institute of Imaging Science on three different 3T Philips Achieva scanners, each with a 32-channel head coil. During each scan session, diffusion-weighted (DWI) and T1-weighted anatomical images were collected. The DWI scans were acquired using a single-shell echo planar imaging sequence (EPI) with TR/ TE = 8600/66 msec and b = 2000 sec/mm^2^in 60 diffusion directions. The data were acquired in 96 x 94 matrices with an isotropic voxel resolution of 2.5 mm^3^. Diffusion weighting was applied along 60 gradient directions evenly distributed across the unit sphere. Acquisition time was 9 min and 6.28 sec per scan. Additionally, six non-diffusion weighted volumes (b = 0) were acquired. The b0 scans were acquired with TR/ TE = 351/3.3 msec. The data were acquired in 80 x 80 matrices with an isotropic voxel resolution of 3 x 3 x 2.5 mm^3^. Acquisition time was 58 sec per scan.

The T1-weighted images were acquired using a gradient recalled echo protocol (MP-RAGE) with TR/TE = 8.9/4.61 msec, flip angle = 8°, and an isotropic voxel size of (1 mm)^3^. Each slice was acquired in a 256 x 256 matrix. The total acquisition time was 4 min and 24.3 sec per scan.

### 2.3 Preprocessing

Preprocessing was performed using QSIPrep 0.16.1 (Cieslak et al., 2021), which is based on Nipype 1.8.5 (K. Gorgolewski et al., 2011;K. J. Gorgolewski et al., 2018); RRID:SCR_002502). Many internal operations of QSIPrep use Nilearn 0.9.2 (Abraham et al., 2014); RRID:SCR_001362) and Dipy (Garyfallidis et al., 2014). For more details of the pipeline, see the section corresponding to workflows in QSIPrep’s documentation (https://qsiprep.readthedocs.io/). The text in the following two sections was automatically by QSIPrep under a CC0 creative commons license to ensure transparency and reproducibility of the preprocessing pipeline.

#### 2.3.1 Anatomical data preprocessing

The T1-weighted (T1w) image was corrected for intensity non-uniformity (INU) using N4BiasFieldCorrection (Tustison et al., 2010; ANTs 2.4.0), and used as T1w-reference throughout the workflow. The T1w-reference was then skull stripped using antsBrainExtraction.sh (ANTs 2.4.0), using OASIS as target template. Spatial normalization to the ICBM 152 Nonlinear Asymmetrical template version 2009c (Fonov et al., 2009; RRID:SCR_008796) was performed through non-linear registration with antsRegistration (ANTs 2.4.0, RRID:SCR_004757,Avants et al., 2008), using brain-extracted versions of both T1w volume and template. Brain tissue segmentation of cerebrospinal fluid (CSF), white matter (WM), and gray matter (GM) was performed on the brain-extracted T1w using FAST (FSL 6.0.5.1:57b01774, RRID:SCR_002823,Y. Zhang et al., 2001).

#### 2.3.2 Diffusion data preprocessing

MP-PCA denoising as implemented in MRtrix3’s dwidenoise (Veraart et al., 2016) was applied with a five-voxel window. After MP-PCA, B1 field inhomogeneity was corrected using dwibiascorrect from MRtrix3 with the N4 algorithm (Tustison et al., 2010). After B1 bias correction, the mean intensity of the DWI series was adjusted so all the mean intensity of the b = 0 images matched across each separate DWI scanning sequence.

FSL (version 6.0.5.1:57b01774)’s eddy was used for head motion correction and eddy current correction (Andersson & Sotiropoulos, 2016). Eddy was configured with a q-space smoothing factor of 10, a total of 5 iterations, and 1000 voxels used to estimate hyperparameters. A linear first level model and a linear second level model were used to characterize eddy current-related spatial distortion. q-space coordinates were forcefully assigned to shells. Field offset was attempted to be separated from subject movement. Shells were aligned post-eddy. Eddy’s outlier replacement was run (Andersson et al., 2016). Data were grouped by slice, only including values from slices determined to contain at least 250 intracerebral voxels. Groups deviating by more than 4 standard deviations from the prediction had their data replaced with imputed values. Final interpolation was performed using the jac method.

Based on the estimated susceptibility distortion, an unwarped b = 0 reference was calculated for a more accurate co-registration with the anatomical reference. Several confounding time series were calculated based on the preprocessed DWI: framewise displacement (FD) using the implementation in Nipype (following the definitions byPower et al. (2014)). The head motion estimates calculated in the correction step were also placed within the corresponding confounds file. Slicewise cross-correlation was also calculated. The DWI time series were resampled to ACPC, generating a preprocessed DWI run in ACPC space with 2.5 mm isotropic voxels.

### 2.4 Tractometry with pyAFQ

After preprocessing the diffusion data, we used pyAFQ 1.3.2 (Kruper et al., 2021) to perform tractography and calculate tractometry metrics for each participant. Fiber orientation distributions (FOD) were estimated in each voxel using constrained spherical deconvolution implemented in DIPY (Garyfallidis et al., 2014;Tournier et al., 2007). After generating FODs, probabilistic tractography was used to generate 200,000 streamlines in the white matter. From these streamlines, 22 major white matter tracts were identified using the approach originally outlined byYeatman et al. (2012). Once identified, each tract was sampled to 100 nodes and fractional anisotropy (FA), mean diffusivity (MD), radial diffusivity (RD), and axial diffusivity (AD) were calculated at each node using a diffusion tensor model (DTI). These tract profile data were then used to examine the relationship between white matter development and academic skills.

### 2.5 Quality control

To ensure that only high-quality neuroimaging data were used in our statistical analyses, we utilized*dmriprep-viewer*to generate quality control scores from the visual reports generated by QSIPrep (Richie-Halford et al., 2022a,2022b). This interactive tool allows raters to generate QC ratings based on various aspects of the preprocessed imaging data, including the diffusion acquisition time series, motion parameters over the course of the diffusion acquisition, the brain mask and the b = 0 to T1w registration, and a 3-d color-coded FA map overlaid on the b = 0 image.

For the present data, two independent raters first completed a training on how to assess the quality of dMRI data using*dmriprep-viewer*before evaluating the same sub-set of scans (N = 174 scans) to ensure reliability across QC ratings. There was 79% agreement in the QC scores given by the two raters in this sub-set. After this initial round of QC, the raters discussed the scans they did not agree upon to establish a common understanding for how to score images. QC scores were determined using a pass/fail criterion that involved assessing movement within the DWI scan, visually inspecting images for artifacts, examining slice dropout during preprocessing, and evaluating the adequacy of the FA map of each scan acquisition. After this discussion, the remaining 526 scans were divided into half and reviewed by each rater. Overall, 128 scans did not pass quality control, leaving a final sample of 572 quality scans across all 4 time points to be used for analysis. This quality control procedure left us with a final sample of 101 participants who had completed either 3 or 4 observations with both behavioral and neuroimaging data.

### 2.6 Data harmonization

The diffusion MRI data used in this study were collected using three different scanners. To account for potential variation between the scanners, we performed ComBat harmonization (Fortin et al., 2017,^2018^;Johnson et al., 2007) on the tract profile data, while protecting potential effects of age and reading ability. We did not protect for mathematics ability, as many participants did not complete mathematics assessments at various time points and would, therefore, be dropped from the harmonization model due to incomplete data. To ensure that these ComBat models did not overfit the data, we fit our models using a fivefold cross-validation scheme, where all of a participant’s data across all time points were included in the same train/test split. This process removes any potential non-biological effects due to scanner differences that might confound subsequent analysis of the tract profile data (Supplementary Fig. S3).

### 2.7 GAMM modeling

We began our analysis by fitting a series of generalized additive mixed models (GAMMs;Hastie & Tibshirani, 1987;Lin & Zhang, 1999;Wood, 2011,^2017^) to model white matter development along the length of an entire tract. Briefly, GAMMs make use of non-linear smoothing functions to flexibly capture non-linearities in the data, while also allowing for the inclusion of both random effects and parametric fixed effects. These models are especially effective at capturing non-linearities in both space and time, which makes them well suited for modeling the longitudinal tract profile data generated by pyAFQ. In the present analysis, we fit a series of GAMMs modeling the development of mean diffusivity at each node along the length of a given tract.

In our GAMM examining the link between white matter development and reading, we included initial age, sex, and three imaging quality control metrics. We also included smoothing terms on time elapsed since the first observation, position on the tract, average reading scores across time points (Reading-Trait), within-participant centered reading (Reading-State), and smooth interactions between tract position and both reading-state and time elapsed since study onset. This model also included random effects of participant, time, and position along the tract to allow for individual differences in overall MD, the rate of MD development, and the variation in MD along the length of the tract.

We also incorporated an AR(1) model in our GAMM (with ⍴ = 0.965) to account for the spatial autocorrelations present in tract profile data (Van Rij et al., 2019). The value of ⍴ was determined using the procedure outlined inVan Rij et al. (2019), whereby we fit our model without any autocorrelation structure and then calculated the ACF lag score of the model. We fit this model in both the left and right arcuate to test whether a potential link between growth in reading and white matter development was specific to the left arcuate or a more global phenomenon found throughout the white matter. The GAMMs used in this analysis were specified as follows:

MDtni=β0+β1(InitialAgei)+β2(Sexi)++β3(NeighborhoodCorrti)+β4(NumBadSlicesti)+β4(MaxRelTranslationti)   +F1(TimeInStudyti)+F2(Noden)+F3(ReadingTraiti)+F4(ReadingStateti)+F5(Noden*ReadingStateti)   +F6(Noden*TimeInStudyti)+u0i+u1i(TimeInStudyti)+u2ni+ε,

where the β coefficients represent the average relationship between the parametric predictors and mean diffusivity at node*n*at time point*t*. Our primary predictors of interest are the smooth functionsℱ*_3_*andℱ*_4_*.ℱ*_3_*tests the hypothesis that overall reading skill is linked with mean diffusivity, whereasℱ*_4_*tests the hypothesis that within-individual changes in reading are linked to changes in the white matter. The functions${ℱ}_{i}$represent non-parametric smoothing terms that allow for non-linear relationships between mean diffusivity and the inputs to those functions.$u_{0i}$and$u_{1i}$represent individual intercepts and growth rates, respectively, and$u_{2ni}$represents individual deviations from the prototypical tract profile. Finally,εis an error term representing any variance in mean diffusivity not captured by the model.

To ensure a parsimonious model, we first constructed a GAMM only capturing growth before sequentially adding the reading predictors to the model. At each step, we compared the full and reduced models using a$\chi^{2}$test for nested models and included the predictor only if this test resulted in p-value of less than 0.05. Furthermore, we assessed the adequacy of the number of basis dimensions in each smooth term by examining the significance of the smooth terms of the GAMM predicting the residuals of our proposed model. None of these smooth terms emerged as significant, indicating that the smoothers in our final model are sufficiently “wiggly” and adequately capturing the variance in the non-linear relationships in the data. Model predictions and standard error estimates were generated using the*predict.gam*function included in the*mgcv*package.

### 2.8 Linear modeling

To examine the longitudinal relationship between the development of the left arcuate and reading development, we fit a linear-mixed effects model predicting mean-centered reading scores, as outlined inRoy et al. (2024). This model included fixed effects of time point, initial age, within-individual mean-centered mean MD in the left arcuate at each time point (MD-state), and overall mean MD in the left arcuate across all time points (MD-trait). This model also included participant-specific random intercepts and slopes on time point to allow for interindividual differences in initial reading scores and growth in reading over time. In addition to this model, we also fit a similar model using a continuous measure of time (time elapsed since the participants initial visit). These models were specified as follows:

CenteredReadingti=β0i+β1i(SessionIDti)+β2(InitialAgei)                  +β3{MDStateti)+β4{MDTraiti)+eti,

where each participant’s reading score at time*t*is modeled as a function of a participant-specific intercept ($\beta_{0i}$), a participant-specific slope ($\beta_{1i}$), group effects of initial age ($\beta_{2}$), MD-state ($\beta_{3}$), MD-trait ($\beta_{4}$), and a residual error term ($e_{ti}$). The participant-specific parameters,$\beta_{0i}$and$\beta_{1i},$were modeled as

$$\beta_{0i}=\gamma_{0}+\upsilon_{0i}$$

$$\beta_{1i}=\gamma_{1}+\upsilon_{1i,}$$

where$\gamma_{0}$and$\gamma_{1}$refer to the average initial reading score and average rate of reading development, respectively, and$\upsilon_{0i}$and$\upsilon_{1i}$describe the extent to which each individual differs from these averages.

### 2.9 Modeling time series with mlVAR

To model the relationship between the time series of changes in mean diffusivity in the left arcuate and changes in reading scores, we fit a multi-level vector autoregression model (Bringmann et al., 2013;Epskamp, Waldorp, et al., 2018). Briefly, this model leverages the time series data of average mean diffusivity and reading scores to model the temporal dynamics of these two change processes, while accounting for individual differences through random effects structures. From these time series, we can then determine the extent to which reading measures at one time point predict future changes in the white matter and vice versa.

## 3 Results

### 3.1 Within-individual changes in reading skill track changes in the white matter properties of the left arcuate

We fit a series of generalized additive mixed models (GAMMs) to understand how the longitudinal link between reading and white matter development unfolds over the length of the left arcuate. We first visually examined the longitudinal development of four different diffusion metrics calculated by pyAFQ in the left arcuate fasciculus.Supplementary Figure S1shows how, on average, tract profiles for each diffusion metric develop over time. For the sake of consistency with past studies (Huber et al., 2018), we focus on MD as our main diffusion metric of interest.

Our model predicting MD development in the left arcuate included parametric terms for initial age, sex, and three imaging quality control metrics, and smooth terms on mean reading scores across all observations (reading trait), within-individual changes in reading scores (reading-state), position along the nodes, time in the study, and node by reading-state and node-by-time interaction smooths. Reading-state and trait were determined using Woodcock–Johnson W scores in Basic Reading (see Methods for overview on Woodcock–Johnson W scores). This model also included a random intercept for each participant and random slopes of node and time, allowing both the tract profile and developmental trajectory of the tract to vary for each participant. Time was operationalized as the amount of time elapsed (in years) from the onset of the experiment. See Methods section for overview of model selection procedure and evaluation of model fit.

**Fig. 1.**
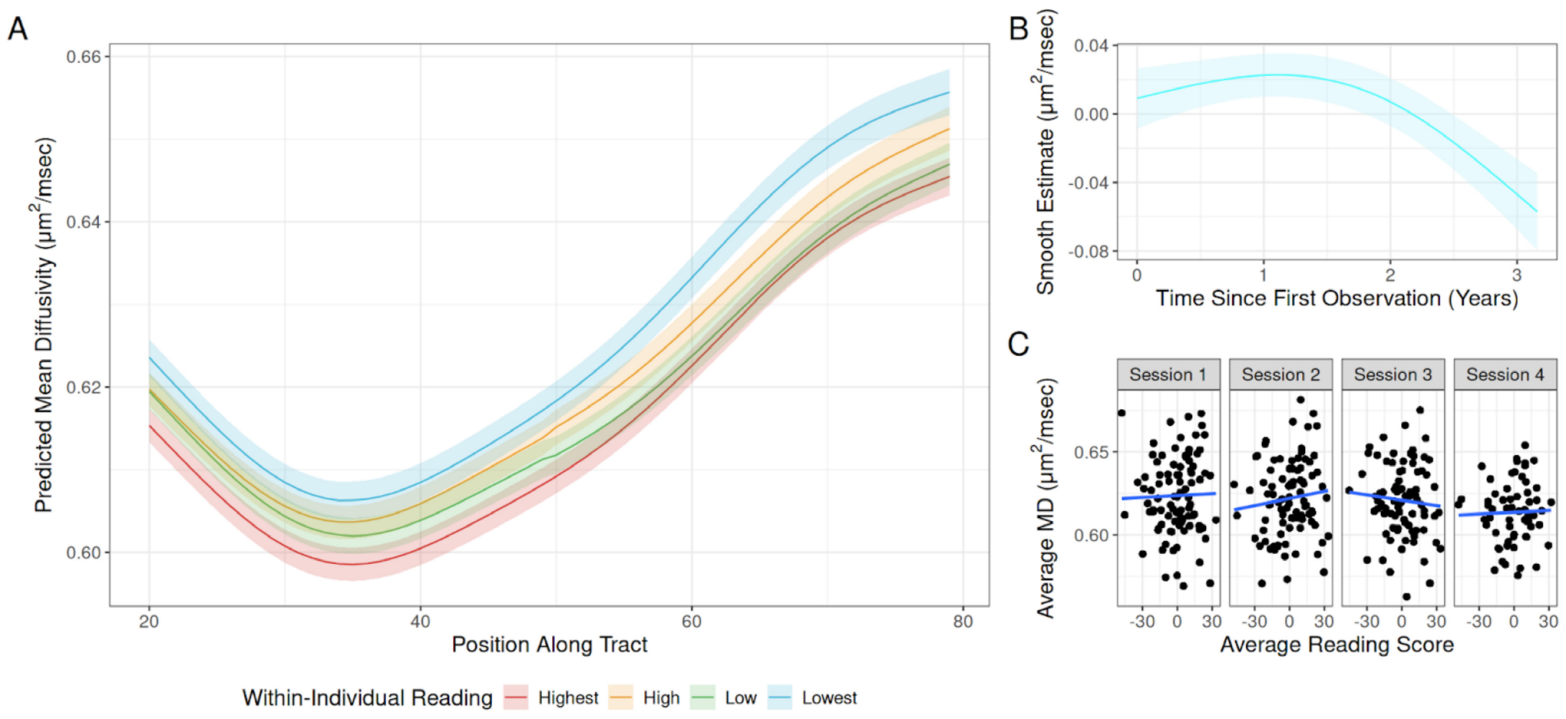
(A) Average estimated tract profiles for MD in the left arcuate fasciculus generated by the GAMM for four different quartiles of reading score change (reading state). Each color represents the magnitude of change relative to the average individual reading score. Shaded areas represent the standard errors of the predictions. (B) The estimated smoothing effect of time elapsed since the first study observation on average MD in the left arcuate. (C) Relationship between overall mean Woodcock–Johnson reading scores and MD in the left arcuate at each time point in the study.

The full output of this model is reported inTable 1. The smooth terms of the model suggest that, on average, within-individual gains in reading are related to changes in MD in the left arcuate, while controlling for initial age, sex, image quality, and mean reading score (F(6.27) = 1.97, p = 0.002;Fig. 1A). There was no observed relationship between mean reading scores and MD (F(1.00) = 0.237, p = 0.626;Fig. 1C). The smooth terms of the model revealed a significant non-linear relationship between node (i.e., location on the tract) and MD (F(8.99) = 279.65, p < 0.001), indicating that MD varies over the length of the left arcuate. Additionally, the smooth term on time revealed a significant non-linear relationship between time and MD (F(1.972) = 6.082, p = 0.001). Visual inspection of the smooth estimate suggested that MD tended to decrease over time in a non-linear fashion (Fig. 1B), in line with expectations from other developmental studies (Lebel et al., 2012;Moura et al., 2016).

**Table 1.** Summary of parametric coefficients and smooth terms for the final GAMM modeling the development of mean diffusivity (MD) across the length of the left arcuate.

Component	Term	Estimate	Std. Error	t-value		p-value
Parametric Coefficients	(Intercept)	5.84E-04	4.01E-05	14.557		<2e-16
	Initial Age	-3.26E-06	5.09E-06	-0.639		0.5226
	Sex	8.13E-06	4.29E-06	1.893		0.0584

Component	Term	edf	Ref. df	F-value	p-value	
Smooth Terms	s(Reading Trait)	1.005	1.005	0.225	0.6349	
	s(Reading State)	1.891	1.97	5.976	0.0018	**
	s(Time)	1.91	1.97	5.888	0.0021	**
	s(Node)	8.838	8.991	279.71	<2e-16	***
	ti(Node, Reading State)	10.884	13.191	3.092	0.0001	***
	ti(Node, Time)	8.427	10.706	1.967	0.0283	*

The model also accounted for image quality metrics.

Signif. codes: 0 <= ‘***’ < 0.001 < ‘**’ < 0.01 < ‘*’ < 0.05.

Adjusted R-squared: 0.823, Deviance explained 0.825.

fREML: -221440, Scale est: 1.000, N: 19260.

Interestingly, we also observed significant smooth interaction between within-individual reading and position along the tract (F(13.224) = 3.126, p < 0.001;Fig. 1A) and between time and position along the tract (F(10.771) = 1.991, p = 0.026). These interactions suggest that the observed relationships between MD and both time and within-individual reading and MD vary over the length of the tract.

Additionally, we fit a reparameterized GAMM using overall age in place of time in study as our primary measure of time. This model revealed a significant smooth effect of age but no main smooth effect of reading state. However, this model did reveal a significant interaction between position along the tract and reading state, similar results to the model reported above (Supplementary Table S3).

To test that the observed relationship between within-individual changes in reading score and changes in the white matter was specific to reading growth and not due to broader maturational changes, we fit the same GAMM to model the development of the right arcuate, a tract not typically associated with reading skill (Vandermosten et al., 2012). This model did not reveal a relationship between MD and either of the smooth terms fit on mean reading scores and within-individual reading (Supplementary Table S1;Supplementary Fig. 6B; both p > 0.05).

However, this model did reveal a significant non-linear relationship between time in the study and MD development (F(1.10) = 6.05, p = 0.010). Visual inspection of this smoothing term again suggested that, on average, MD decreases as a function of time (Supplementary Fig. S6C). Furthermore, the smooth terms of this model revealed significant non-linear interactions between position along the tract and both time in study (F(4.42) = 3.030, p = 0.016) and within-individual reading (Supplementary Fig. S6A; F(11.60) = 3.44, p < 0.001), suggesting that the relationship between MD and both reading and time varies over the length of the tract.

Additionally, we fit a model predicting MD development in the left arcuate from mathematics scores to test that the observed relationship between reading and MD development was specific to reading. To do so, we fit the same GAMM using mean mathematics scores and within-individual changes in Woodcock–Johnson W scores in mathematics, instead of reading scores. The full results are presented inSupplementary Table S2, but briefly, we found no significant relationships between either overall mathematics score or within-individual changes in mathematics scores and MD in the left arcuate (both p > 0.05).

### 3.2 White matter properties and reading skill, but not mathematics, demonstrate between-individual variance in development over a 4-year period

We then sought to understand why we observed a longitudinal relationship between the development of the left arcuate and growth in reading, but not mathematics, despite observing a relationship between reading and math skill at each time point (Supplemental Figure 5). If there is not significant variance in the growth rates of either white matter maturation or academic skills, then we cannot expect to see a longitudinal, within-individual relationship between these factors. To examine interindividual differences in growth trajectories of academic skills, we fit a series of nested linear growth models (Grimm et al., 2016) modeling the growth of either Woodcock–Johnson W scores in Basic Reading or Woodcock–Johnson W scores in mathematics. To identify between-individual differences in the growth trajectories of the MD tract profiles in the left arcuate, we again relied on GAMMs instead of linear mixed effects models.

**Fig. 2.**
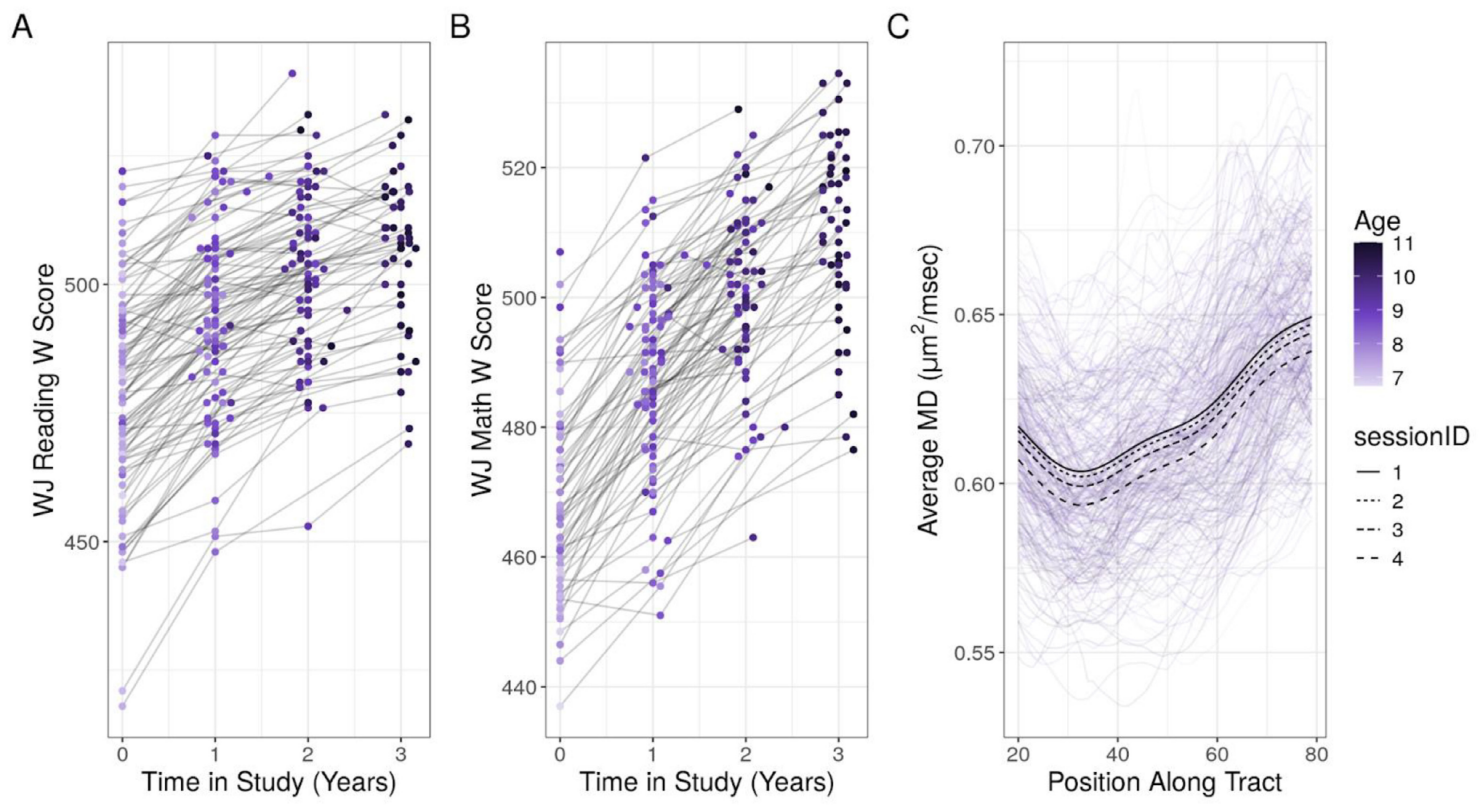
Individual growth trajectories of Woodcock–Johnson composite reading (A) and mathematics (B) W scores. Each line represents an individual participant and the color of each dot represents the participant’s age at a given observation. (C) Within-individual changes in MD in the left arcuate fasciculus. Each purple profile represents a given participant at a given scan session. The black lines represent the average estimated tract profile at each time point in the study.

In the case of reading, the addition of a term capturing participant-specific developmental trajectories significantly improved the model fit compared with a reduced model that included a random intercept but not a random slope (χ^2^(2) = 19.738, p < 0.001). This finding indicates that there is significant variation in how individuals’ reading scores change over time (Fig. 2A). Interestingly, this was not the case for Woodcock–Johnson W mathematics scores, where the addition of a participant-specific developmental slope did not improve the model fit compared with a reduced model (χ^2^(2) = 0.969, p = 0.616;Fig. 2B), suggesting that the participants in this study progressed in mathematics at roughly the same rate.

To understand between-individual differences in MD development in the left arcuate, we fit a GAMM that included a smooth term over the length of the tract, as well as random effects of participant, position along the tract, and time elapsed since the first observation. The addition of a random slope on time significantly improved the model fit (F(8) = 8.165, p < 0.001), suggesting that there are significant between-individual differences in how MD in the left arcuate changes over time (Fig. 2C;Supplementary Fig. S2).

### 3.3 Longitudinal changes, not cross-sectional differences, in reading are related to white matter development in the left arcuate fasciculus

After identifying a relationship between the within-individual development of both reading and MD in the left arcuate, we also looked to replicate the findings reported inMeisler and Gabrieli (2022)andRoy et al. (2024)that suggest that static properties (i.e., cross-sectional correlations at a given time point) of the left arcuate are not related to reading skill. When we examined the correlation between average MD across the length of the tract and reading skill at each time point, we found no significant relationship between reading scores and MD at any of the four observations (all r < 0.12, all p > 0.05;Fig. 1C).

We also looked to replicate the finding reported inRoy et al. (2024), suggesting that within-individual changes in reading skill are related to within-individual changes in the mean values of the diffusion properties of the left arcuate. To do so, we calculated the average MD (MD_avg_) of the left arcuate for each participant at each time point and then fit a linear mixed effects model predicting mean-centered reading using time point, initial age, image quality metrics, mean-centered MD_avg_, and MD_avg_across time (state- and trait-MD_avg_, respectively) as fixed effects and time point and participant as random effects.

**Fig. 3.**
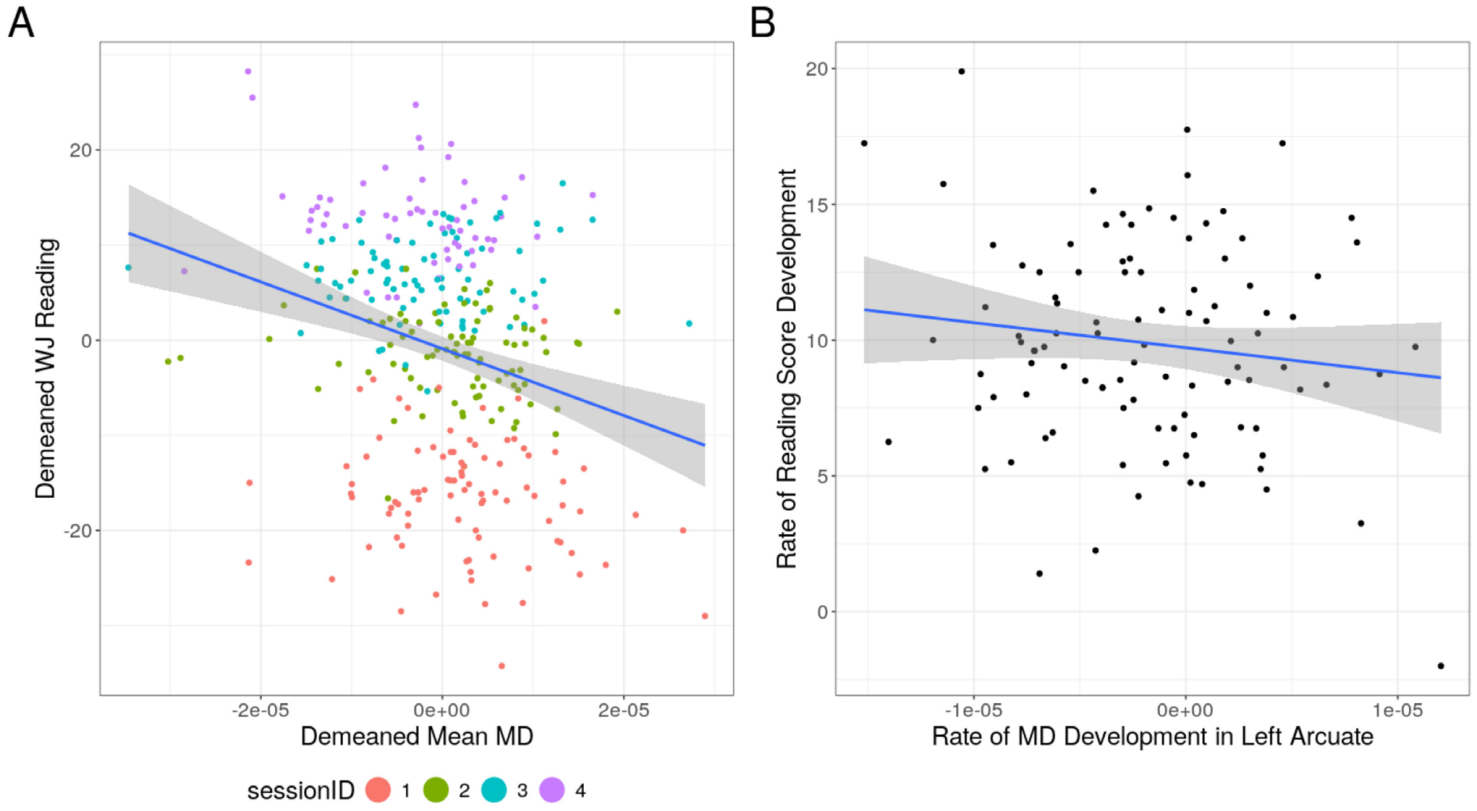
(A) Relationship between within-individual mean-centered Woodcock–Johnson reading W scores (y-axis) and within-individual mean-centered mean diffusivity in the left arcuate fasciculus. The color of each dot represents the study session. (B) Relationship between individual rates of reading development (y-axis) and rate of mean diffusivity (MD) change in the left arcuate (x-axis).

This model revealed significant effects of time point (t(314) = -27.387, p < 0.001), and initial age (t(314) = -2.386, p = 0.018), indicating that, as expected, reading scores increase over time. Mean-centered MD_avg_was also a significant predictor of reading scores (t(314) = -2.007, p = 0.046;Fig. 3A), indicating that session-to-session changes in MD predict changes in reading score above and beyond the progression of time. Furthermore, there was no significant relationship between MD_avg_and mean reading (t(314) = 1.687, p = 0.092;Fig. 3C). In addition to this model, we also fit a similar linear mixed effects model using time elapsed since the start of the study as a continuous measure of time, instead of discrete session numbers. Although the coefficients were largely consistent across the two models, the relationship between mean-centered MD_avg_and mean-centered reading was not statistically significant in the model that included a continuous measure of time (t(311) = -1.642, p = 0.1016).

We then examined the relationship between growth rates in reading and growth rates in MD_avg_within the left arcuate. To estimate growth rates, we followed the approach outlined inYeatman et al. (2012)and fit linear models for each participant predicting either reading skill or MD_avg_as a function of time elapsed from the first observation. We then examined the relationship between the rate of MD_avg_development and reading development by calculating the correlation between the individual growth rates. We observed a significant relationship between the rate of reading growth and MD_avg_development in the left arcuate (r = -0.12, p = 0.0314;Fig. 3B), suggesting that the rate at which these changes occur is related to one another. Additionally, we examined the variation in MD_avg_growth rates in relation to average reading scores across all time points to determine whether good versus struggling readers show different patterns of MD_avg_change in the left arcuate. This analysis revealed no relationship between average reading scores and MD_avg_development (r = 0.015, p = 0.80;Supplementary Fig. S4).

### 3.4 Gains in reading precede white matter development across the left arcuate

**Fig. 4.**
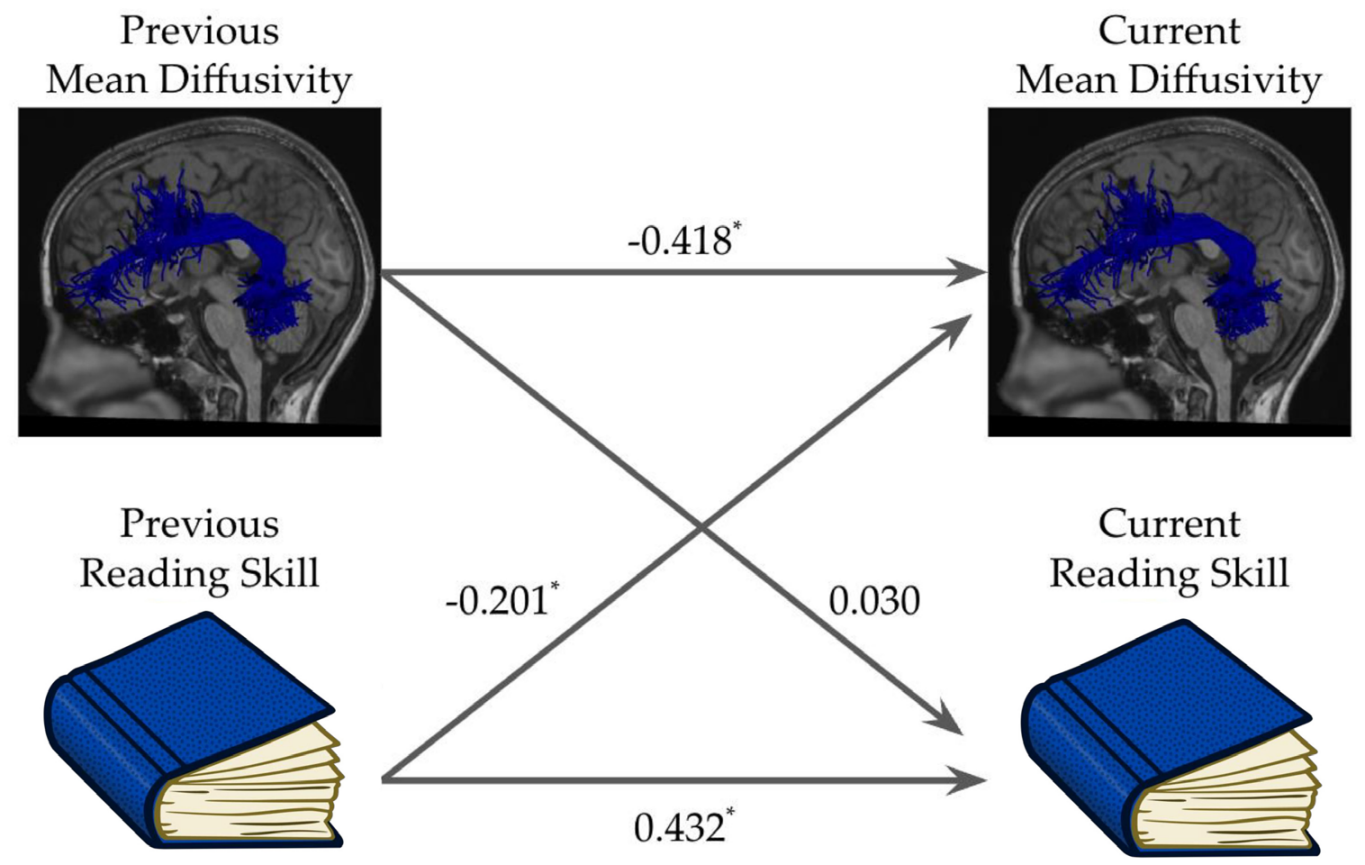
Path diagram illustrating the mlVAR model examining the relationship between the time series of reading development and MD_avg_in the left arcuate. The values along each path represent the beta-weights estimated by the model. All paths were significant, with the exception of the path of previous MD_avg_predicting current reading skill.

Given the observed longitudinal relationship between changes in reading scores and changes in MD_avg_in the left arcuate, we then modeled the relationship between the time series of individual reading development and individual white matter development. To do so, we fit the same multi-level vector auto-regression model (mlVAR;Bringmann et al., 2013;Epskamp et al., 2018) used inRoy et al. (2024). Briefly, these models use the time series of reading scores and white matter development to examine whether growth in one variable predicts future growth in the other, while accounting for between-individual differences through random effects structures. This model revealed that higher reading scores at one time point were associated with lower MD_avg_at the subsequent time point (t(215) = -2.984, p = 0.007;Fig. 4), whereas lower MD_avg_did not predict future reading scores (t(215) = 0.411, p = 0.565). A bootstrapped difference test (Epskamp, Borsboom, et al., 2018) comparing these two paths revealed that the 95% confidence interval around the difference between these two path weights did not include 0 (95% CI: [0.389, 0.406]), suggesting that these coefficients are significantly different from each other.

## 4 Discussion

In the present study, we leveraged the flexibility of generalized additive mixed models (GAMMs) to understand developmental change in the left arcuate and its relationship with the development of reading skills in a longitudinal sample of 101 children from the southern United States. Furthermore, we looked to replicate the longitudinal results presented inRoy et al. (2024)in an independent sample and extend them to examine the longitudinal relationship between white matter development and mathematics skill. We employed the same modeling approaches outlined inRoy et al. (2024)to examine the within-individual relationship between white matter development and both reading and mathematics learning.

In the case of reading, we replicated the finding that changes in the diffusion properties of the left arcuate fasciculus correspond to gain in reading over time. At a given time point, we did not observe any cross-sectional relationship between reading skill and white matter properties. However, both the linear and GAMM growth models revealed that within-individual decreases in MD_avg_and node-wise MD correspond with reading gains over time. Through the use of GAMMs, we were able to extend the original results and account for non-linear developmental trends across the entire length of a given tract. We also replicated past findings linking the linear rate of reading change with the linear rate of white matter change in the left arcuate (Roy, Richie-Halford, et al., 2024;Yeatman, Dougherty, Ben-Shachar, et al., 2012). Additionally, through the use of mlVAR models, we replicated the finding that gains in reading predict future development of the left arcuate, but not vice versa. Overall, these findings replicate past findings suggesting a dynamic relationship between reading skill and white matter development that is best viewed through a longitudinal lens.

Additionally, we found that the relationship between MD development and gains in reading varied over the length of the left arcuate. Visual inspection of the node by reading-state interaction suggested that the longitudinal relationship between reading and MD was strongest in the posterior portions of the left arcuate located within the temporal lobe (Fig. 1A). The varying relationship between reading-state and MD over the length of the arcuate is in line with past results that have examined node-wise correlations with reading across the left arcuate (Gullick & Booth, 2015;Yeatman, Dougherty, Myall, et al., 2012). The observed interaction may be related to the proximity between the posterior portion of the left arcuate and ventral occipito-temporal cortex (VOTC), functional activity which has been linked with reading (Dehaene & Cohen, 2011;Perrone-Bertolotti et al., 2014).

Furthermore, this finding highlights the importance of tract profile data, as opposed to mean diffusion metrics collapsed across a tract, for understanding white matter development. Although past work has demonstrated the utility of using mean diffusion metrics to examine the white matter underpinnings of reading and other cognitive skills, the combination of tract profile data and flexible, non-linear modeling approaches, such as GAMs, allows for additional nuance into understanding how brain–behavior relationships play out over the length of a white matter tract. Future studies should consider employing similar methods to understand the white matter correlates of development, behavior, and disease progression.

Interestingly, we also observed a significant node by reading-state smooth interaction in the right arcuate. Although we did not observe a significant main effect of within-individual reading on MD, this interaction suggests that portions of this tract may relate to reading skill, again highlighting the importance of tract profile data. Past studies have observed differences in both functional activation and white matter properties in the right hemisphere that correlate with reading comprehension (Cutting et al., 2013;Horowitz-Kraus et al., 2014), suggesting that some structures in the right hemisphere, such as the right arcuate, may be involved with some aspects of reading. Future longitudinal research using more specific reading measures will be needed to further understand the role of the right arcuate in supporting the development of reading and literacy.

The observed behavioral gains in reading potentially stem from functional changes in the cortical areas responsible for skilled reading. Functional MRI studies have demonstrated reduced activity in the VOTC in struggling readers (Brem et al., 2020;Kubota et al., 2019) and that learning gains in reading are linked to changes in functional activation of VOTC (Heim et al., 2015;Mitchell et al., 2025;Yeatman et al., 2024), as well as a range of other cortical areas (Barquero et al., 2014). Additionally, reading interventions have been found to increase functional connectivity between VOTC and other cortical regions (Horowitz-Kraus et al., 2019). Together these results suggest that learning gains in the domain of reading can drive functional changes in the cortical areas supporting literacy.

These changes in functional activity may, in turn, promote the proliferation of glial cells and/or increases in the myelination of the white matter pathways underlying reading. Evidence from animal models has shown that increases in neural activity can increase both oligodendrogenesis and levels of myelination along specific axonal bundles (Fields, 2015;Gibson et al., 2014), which would serve to partially explain the results of the mlVAR model showing that behavioral gains in reading precede changes in the white matter. Increases in myelination and/or oligodendrogenesis at the cellular level restrict the diffusion of water thereby driving changes in various diffusion metrics, including decreases in MD (Blumenfeld-Katzir et al., 2011;Peters et al., 2019;Zhou et al., 2020). This putative mechanism could serve to explain the observed relationship between within-individual reading development and longitudinal changes in MD.

Altogether, evidence from animal models and fMRI studies suggest that intensive reading interventions could increase functional activity within the developing reading circuitry, driving subsequent changes in the diffusion properties of the white matter connections within these circuits. However, the time scale used in past intervention studies examining changes in functional brain activity does not align well with that reported in the work byHuber et al. (2018), who detected intervention-related changes in both reading skill and in the white matter after just 2 weeks. It is likely that different mechanisms of plasticity are at play over different time scales. Future intervention studies combining functional and diffusion neuroimaging data, coupled with tract profile-based analyses, will be necessary to better understand the temporal dynamics of the functional and structural plasticity underlying gains in reading skill and how these interactions unfold over the length of white matter tracts.

In addition, we also looked to extend the results presented inRoy et al. (2024)by examining the relationship between white matter development and gains in mathematics. We did not observe any significant relationships, either cross-sectional or longitudinal, between patterns of white matter development and growth in mathematics. Although we observed intra-individual growth in terms of overall mathematics scores, we did not observe significant interindividual differences in the growth rates of mathematics scores. Additionally, we did not observe a relationship between within-individual growth in mathematics skill and within-individual changes in white matter properties. This finding is not entirely surprising given the lack of variation in growth trajectories in mathematics scores in the current sample.

This finding, however, does not necessarily rule out a longitudinal relationship between mathematics learning and white matter development but rather may reflect a limitation of the present sample. Intervention and quasi-experimental studies have revealed a relationship between educational experiences in mathematics and both functional and structural changes in the brain (Iuculano et al., 2015;Klein et al., 2019). It could be the case that the children in the current sample have all experienced similar educational environments in the domain of mathematics and, therefore, do not differ significantly in terms of both behavioral gains in mathematics and the underlying white matter changes. However, it could be the case that, at a population level, regional differences in environmental factors, such as educational environment or policy decisions, influence the longitudinal dynamics between academic skills and white matter development (Roy, Van Rinsveld, et al., 2024;Weissman et al., 2023). Future longitudinal work leveraging multi-site consortium data sets will be necessary to understand these dynamics.

It is also possible that a potential link between white matter properties and mathematics skill is obscured by our approach to harmonization of the tract profile data. Due to missingness with our mathematics assessment, we did not protect for mathematics scores when we harmonized the neuroimaging data. Potential variation in the white matter related to mathematics development may have been obscured through the harmonization process, thereby limiting the conclusions we can make about the relationship between properties of the left arcuate and mathematics development.

Furthermore, we limited the scope of our investigation to the left and right arcuate fasciculus, whereas past studies have identified a link between mathematics skill and the frontoparietal connections of the superior longitudinal fasciculus (SLF;Jolles et al., 2016;Matejko et al., 2013;Tsang et al., 2009). It could be the case that the development of mathematics skill tracks changes in the properties of the SLF and not the left arcuate. However, it also bears mentioning that the left arcuate contains functional sub-components of the left arcuate that have been related to mental arithmetic in adults (Grotheer et al., 2019). These functional sub-bundles might be obscured by our tractography approaches that do not make use of functional MRI data. Future work combining functional and diffusion MRI will have to investigate the development of these functional sub-bundles (Meisler et al., 2024) and how they relate to reading and mathematics development.

Although generally we have replicated the broad findings linking longitudinal changes in reading skill to changes in the white matter, there are some differences between the present results and those reported inRoy et al. (2024). The primary difference is that, while the previous analysis focused on fractional anisotropy (FA), the present results center on mean diffusivity as the primary white matter metric of interest. Visual inspection of the session-by-session tract profiles (Supplementary Fig. S1) indicated that, in the present sample, FA in the left arcuate does not change across the four time points, whereas mean diffusivity follows the expected developmental trends (Lebel & Deoni, 2018;Lebel et al., 2019). The observed lack of FA development in the present sample may be partially due to the relatively high b-value (b = 2000) used for the acquisition of the diffusion-weighted images, as higher b-values reduce signal-to-noise ratio. Despite this limitation in the data, examining the color FA maps suggests a reasonable tensor fit for the calculation of our diffusion metrics (Supplementary Fig. S7).

Nevertheless, despite these inconsistencies, both FA and MD are both useful metrics for measuring change in the white matter and have been shown to change over the course of development and in response to academic learning experiences (Huber et al., 2018;Klein et al., 2019;Yeatman et al., 2012). The fact that we observed a longitudinal relationship between reading skill and within-participant changes in MD suggests that gains in reading are accompanied by changes in the diffusion properties of the left arcuate. However, the biological interpretations of the present results are less clear, especially given the surprising lack of developmental change in FA.

Additionally, the present results need to be interpreted within the context of a few limitations of our modeling approach. First, our ComBat-based harmonization approach relied on linear models, whereas our GAMMs allowed for non-linear smoothing terms. This limitation in our approach harmonization does not account for the non-linear shape of each tract or correlated error structure, potentially biasing our harmonized tract profiles. Secondly, due to limitations in the statistical software used to fit the GAMMs, we could only consider an AR(1) model to account for correlated error terms in the tract profile data. Although examination of the ACF plots of the model with and without the AR(1) structure suggests that this structure accounts for a large amount of correlated noise in the data (Supplementary Fig. S8), other autocorrelation models may better account for the autoregressive structure in the data. Future work is needed to better understand how different autocorrelation functions relate to GAMMs of tract profile data. Finally, our mlVAR approach does not allow for the inclusion of covariates, such as sex or age, in the model. It could be the case that demographic or contextual factors are driving the observed temporal relationships between reading and white matter development.

In summary, these results largely replicate past findings indicating that changes in the properties of the arcuate fasciculus are linked longitudinally with gains in reading skill and that gains in reading precede future changes in the white matter. We extend these findings to a new sample, from a new geographic location. Together, these results further support the hypothesis that neuroplasticity and literacy are dynamic processes that are best understood through a framework centered around within-individual change over time. These insights, coupled with those from future learning intervention studies, will hopefully inform the design and implementation of educational practices and policies. By adopting scientifically grounded approaches to literacy instruction, we can hopefully promote the development of robust white matter networks that support reading development across a wide range of learners.

## Supplementary Material

Supplementary Material

## Data Availability

The data used for this study are not publicly available but may be made available upon request and completion of a data use agreement. The preprocessed derivatives were generated using the publicly available qsiprep software package (https://qsiprep.readthedocs.io/en/latest/). The pyAFQ outputs were generated using the publicly available pyAFQ software package (https://tractometry.org/pyAFQ/). All statistical analyses were conducted using the R programming language (version 4.4.1;R Core Team, 2022). Linear mixed effects models were fit using the*lme4*package (Bates et al., 2015), GAMMs were fit using the*bam*function present in the*mgcv*package (S. Wood, 2015) with random effect smoothers, and the mlVAR model was fit using the*mlVAR*package (Epskamp, Borsboom, et al., 2018;Epskamp, Waldorp, et al., 2018). The code used to generate the present analyses and figures can be found athttps://github.com/earoy/longitudinal_read_wm_replication/.
