# Supplementary material for: Development of the arcuate fasciculus is linked to learning gains in reading
Source: Imaging Neurosci (Camb). 2025 Apr 17;3:imag_a_00542. doi: 10.1162/imag_a_00542 (PMC12320015; doi:10.1162/imag_a_00542)
Supplement: Supplementary Material [file imag_a_00542-supp.pdf]

Supplemental Figures

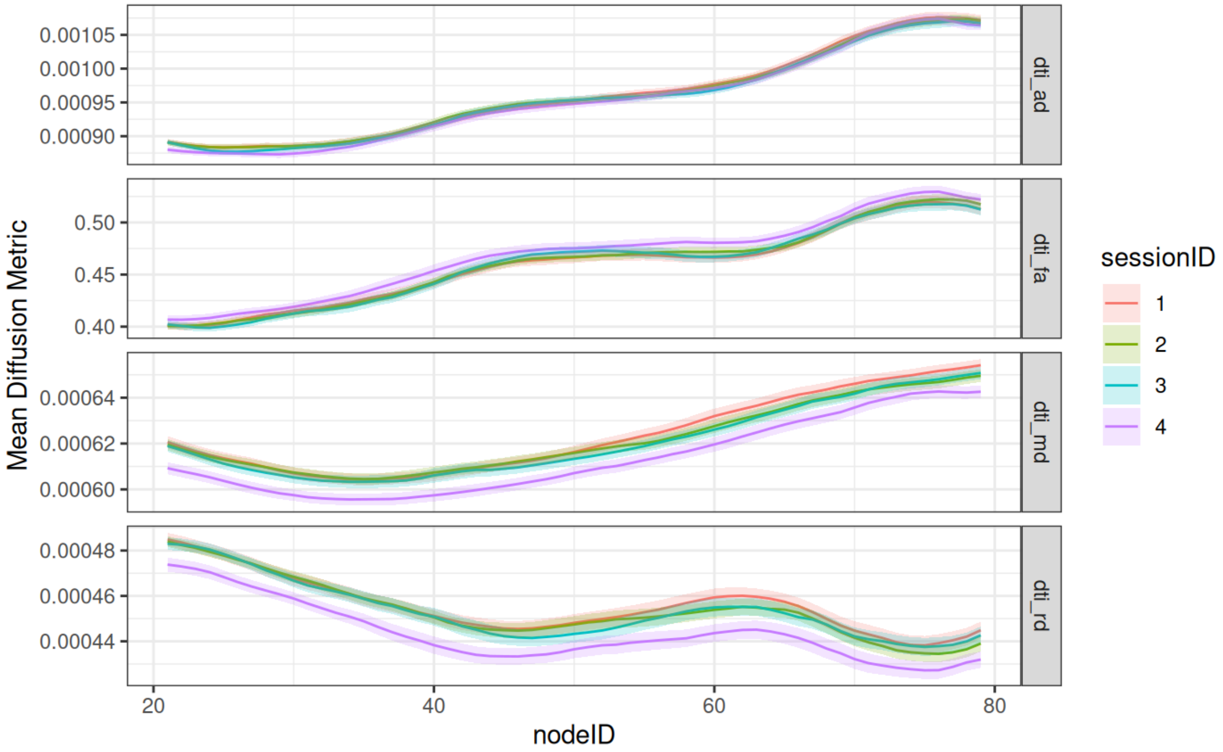

Supplemental Figure 1: From top, tract profiles in the left arcuate for axial diffusivity, fractional anisotropy, mean diffusivity, and radial diffusivity across the four time points of the study.

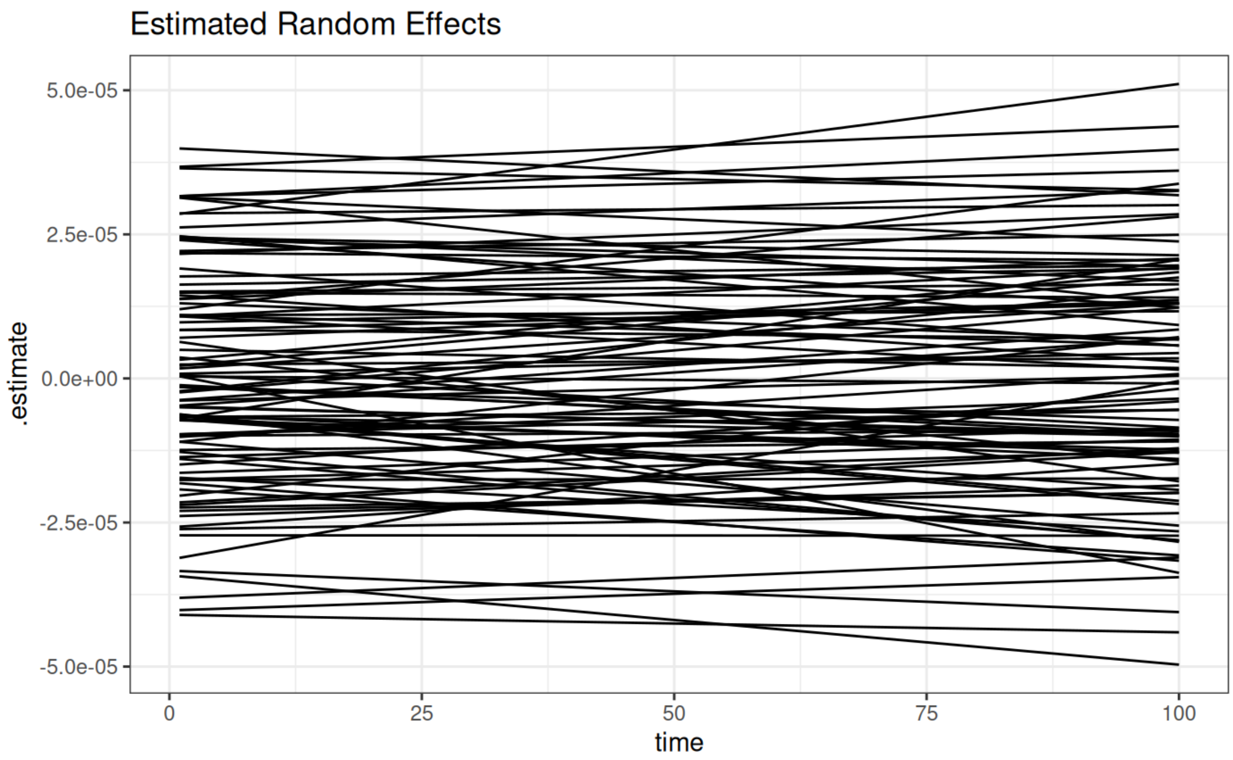

Supplemental Figure 2: Estimated linear growth rates of MD in the left arcuate. Each line represents the estimated average change in MD over time for each participant.

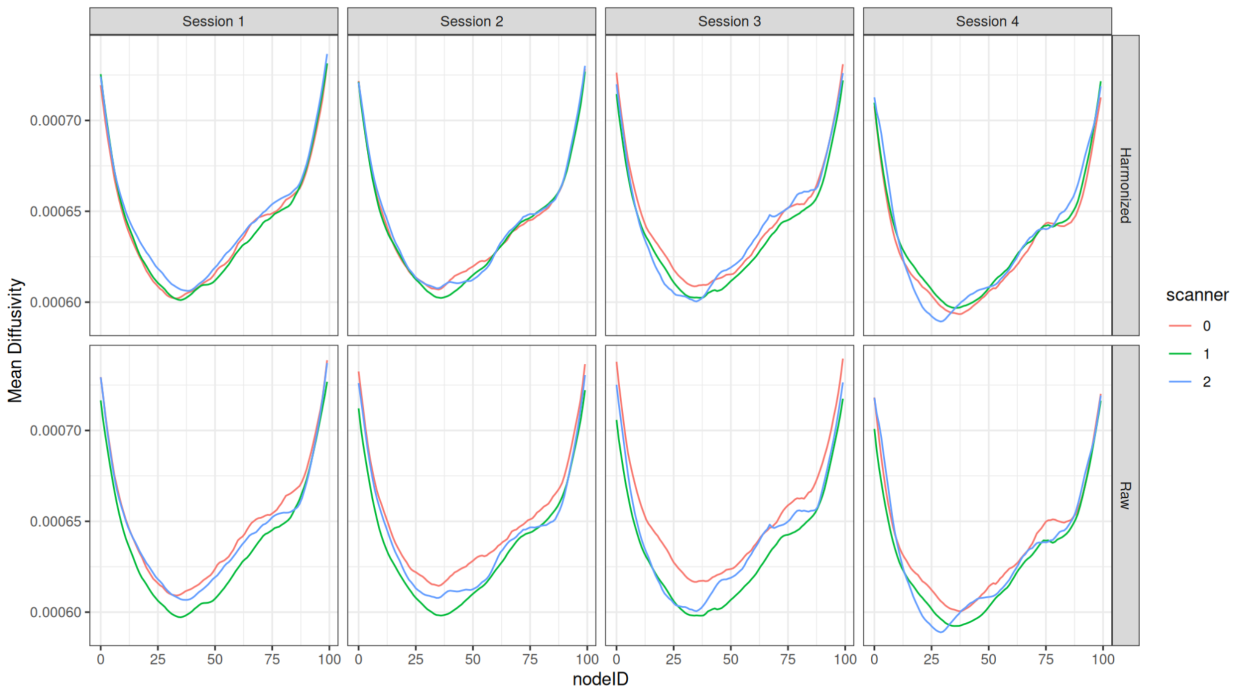

Supplemental Figure 3: Mean tract profiles illustrating mean diffusivity in the left arcuate using raw tract profile data (bottom row) and ComBat harmonized tract profile data (top row) at each time point. Each color represents a different scanner.

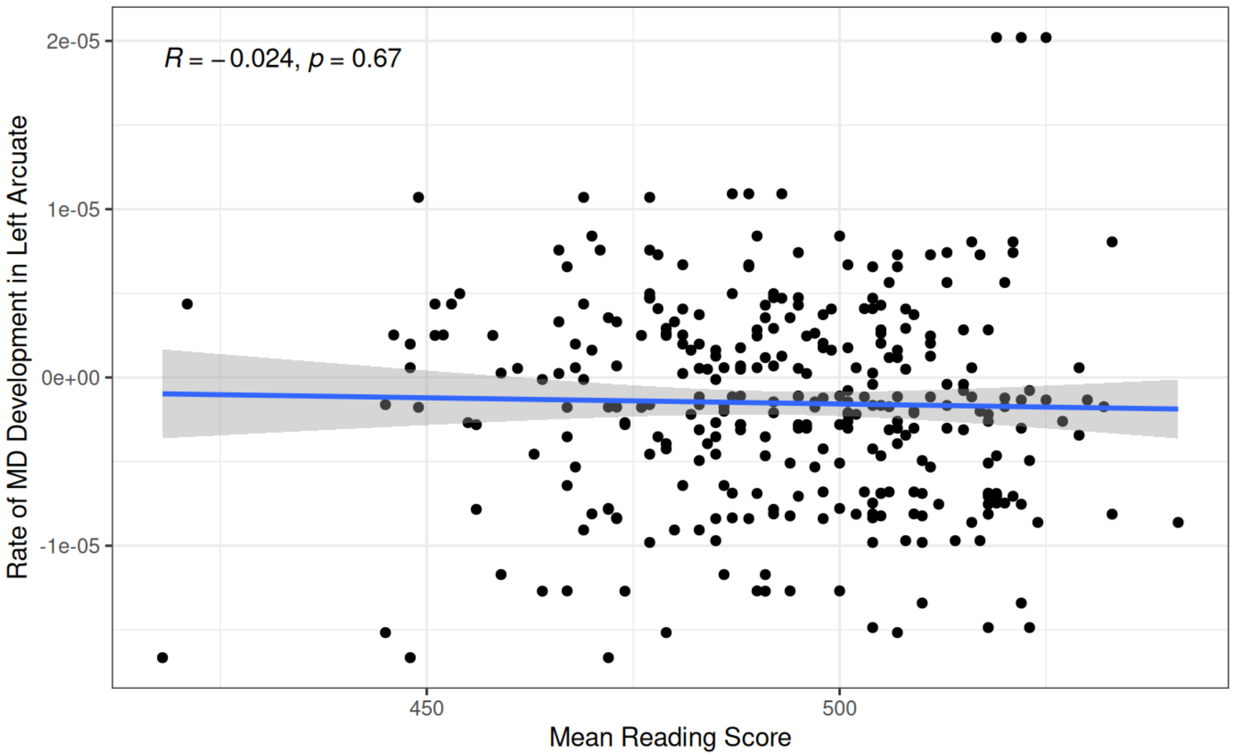

Supplemental Figure 4: Relationship between average reading scores across all study time points and rates of MD development in the left arcuate for each participant.

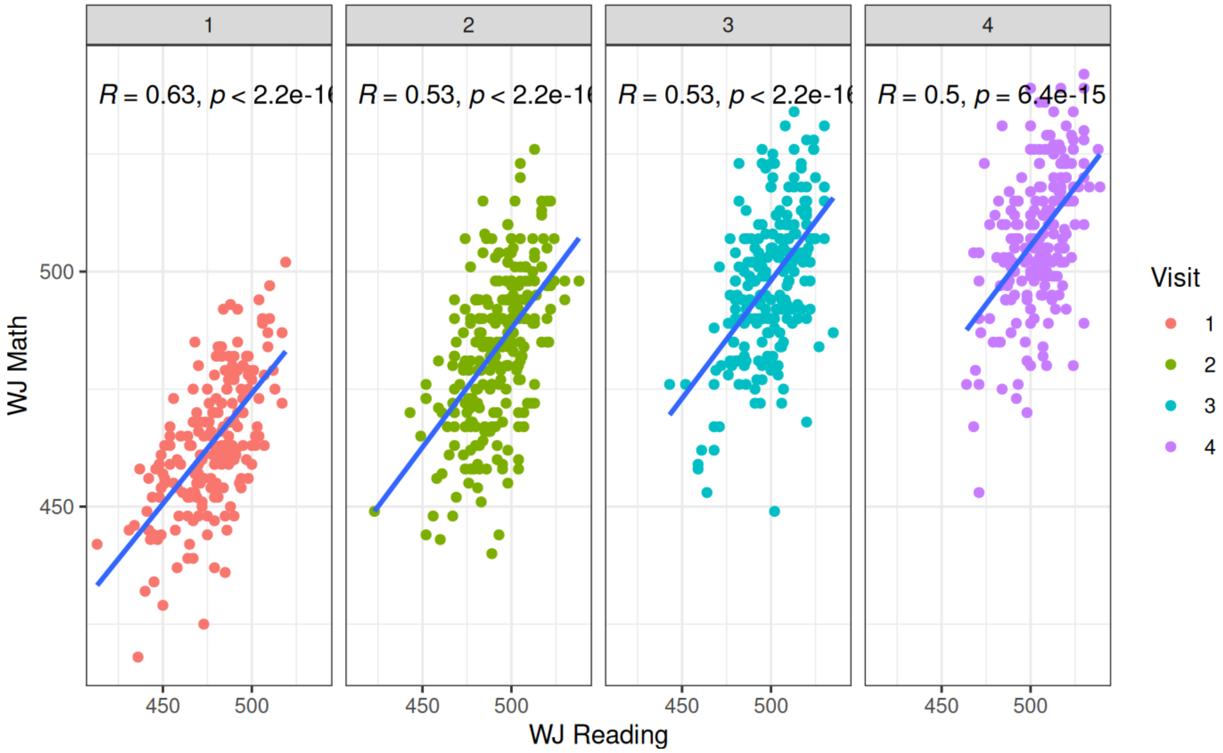

Supplemental Figure 5: Relationship between reading and math scores across all time points. Each point represents an individual at a given time point and colors represent each of the 4 waves of data collection.

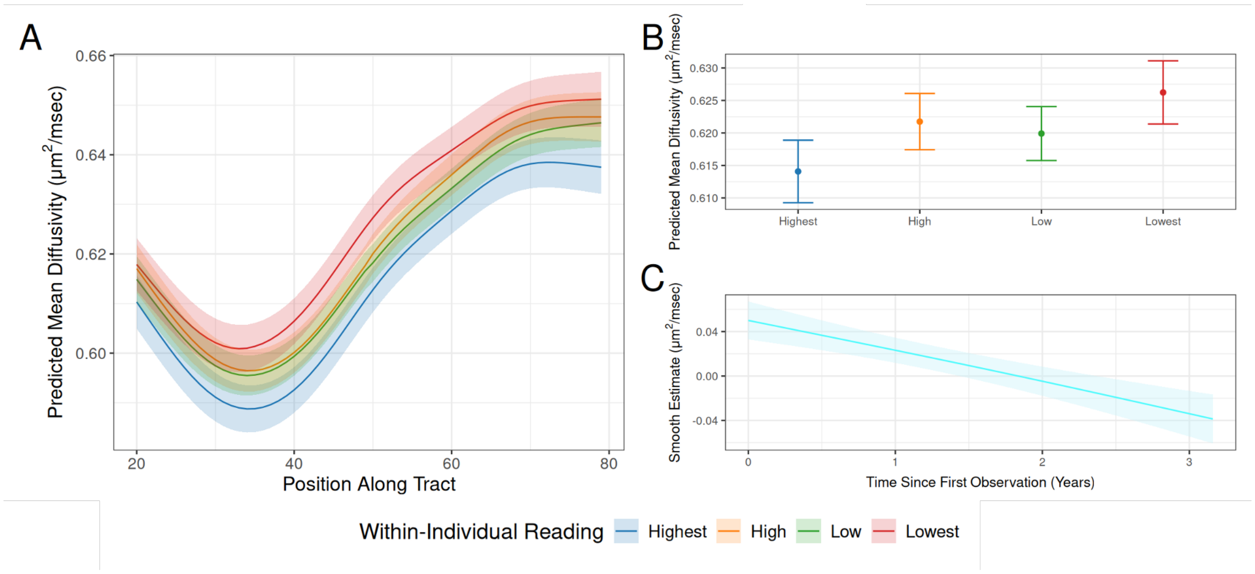

Supplemental Figure 6: A. Average estimated tract profiles for MD in the right arcuate fasciculus generated by the GAMM for four different quartiles of reading score change (reading state). **B.** Relationship between mean MD and reading state in the right arcuate Each color represents the magnitude of change relative to the average individual reading score. **C.** The estimated smoothing effect of time elapsed since the first study observation on average MD in the right arcuate.

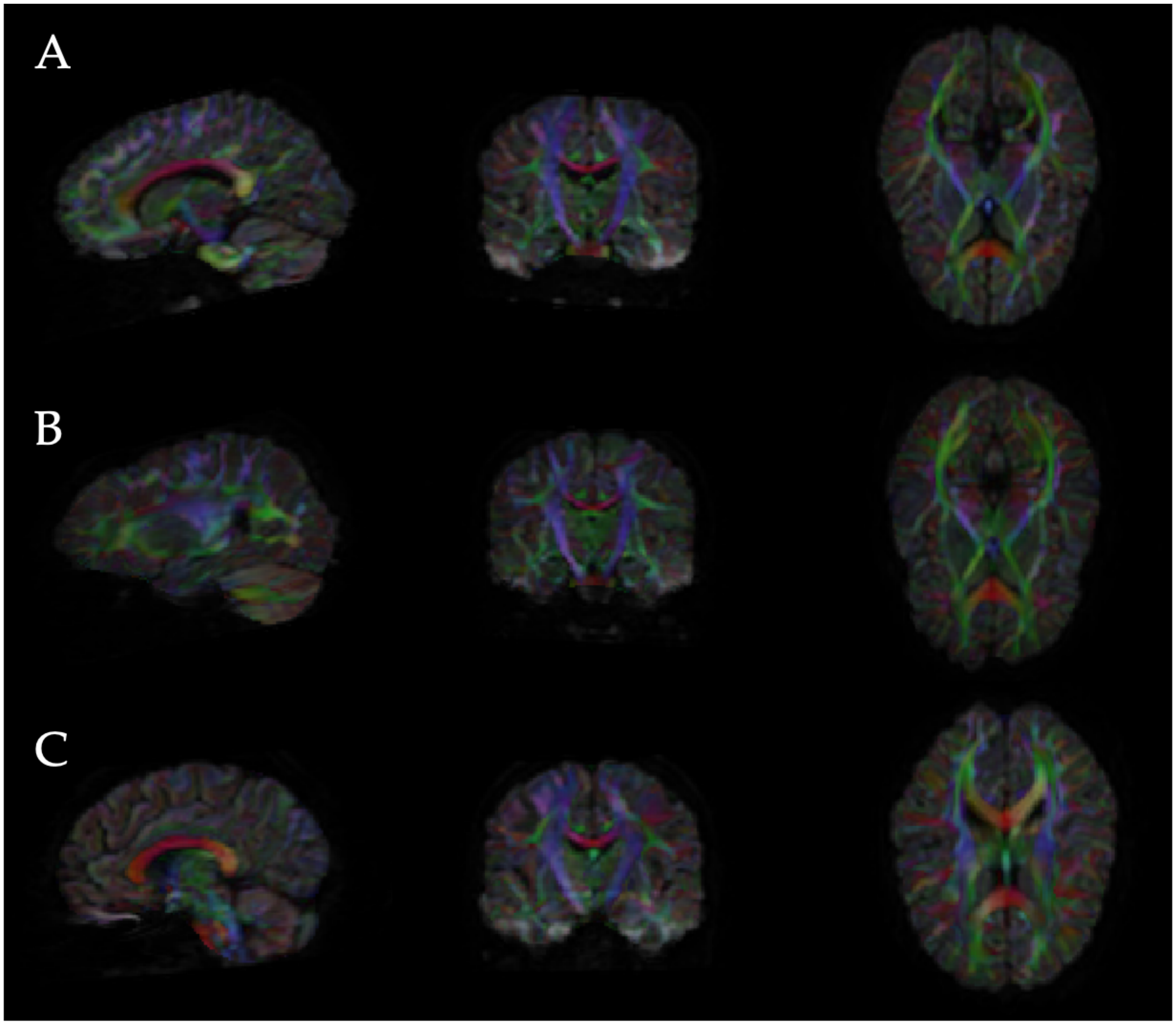

Supplemental Figure 7: Color FA maps viewed in *dmriprep-viewer* for three exemplar participants (A, B, and C). The color refers to the principal direction of diffusion along the x, y, or z axis. Red represents diffusion along the left/right axis, green along the anterior/posterior axis, and blue along the inferior/superior axis.

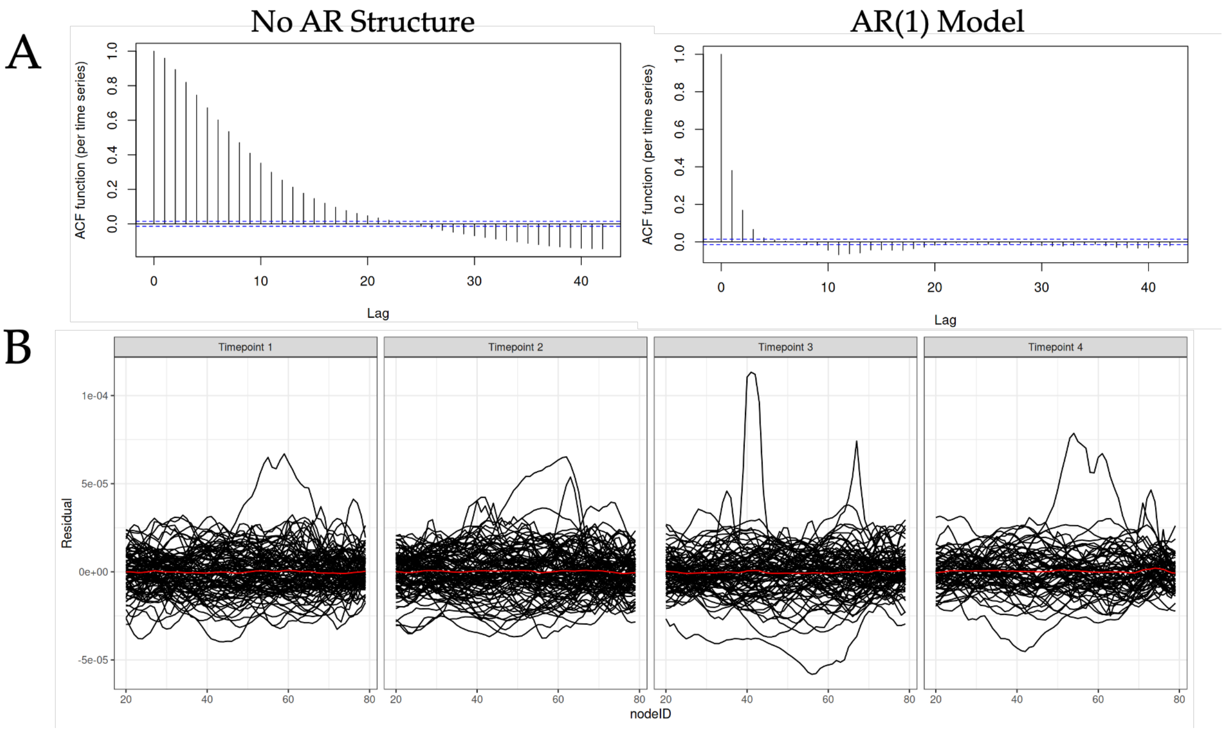

Supplemental Figure 8: A. ACF plots of the residuals for a GAMM model that did not include any autocorrelation structure (left) and a model that included an AR(1) term. **B.** Residual track profiles generated from the final GAMM model, split across all four time points. Each black line represents the participant-level residuals from the model and the red line represents the overall average residual of the model.

Supplemental Table 1: Summary of parametric coefficients and smooth terms for the final GAMM modeling the development of mean diffusivity across the length of the right arcuate.

Component	Term	Estimate	Std. Error	t-value	p-value	
Parametric Coefficients	(Intercept)	5.87E-04	4.06E-05	14.462	< 2e-16	***
	Initial Age	-2.99E-06	5.12E-06	-0.584	0.5593	
	Sex	5.80E-06	4.33E-06	1.340	0.1802	
**Component**	**Term**	**edf**	**Ref. df**	**F-value**	**p-value**	
Smooth Terms	s(Reading Trait)	1.00E+00	1.00E+00	0.135	0.714	
	s(Reading State)	1.79E+00	1.93E+00	2.065	0.184	
	s(Time)	1.13E+00	1.22E+00	5.463	0.011	*
	s(Node)	8.904	8.998	512.209	< 2e-16	***
	ti(Node, Reading State)	9.314	11.646	3.489	< 2e-16	***
	ti(Node, Time)	3.423	4.51	3.116	0.013	*

Signif. codes: 0 <= '*' < 0.001 < '**' < 0.01 < '*' < 0.05**

Adjusted R-squared: 0.82, Deviance explained 0.822

fREML: -218700, Scale est: 1.000, N: 19260

Supplemental Table 2: Summary of parametric coefficients and smooth terms for the final GAMM modeling the development of mean diffusivity across the length of the left arcuate with math scores.

Component	Term	Estimate	Std. Error	t-value	p-value
Parametric Coefficients	(Intercept)	5.75E-04	4.90E-05	11.748	< 2e-16 ***
	Initial Age	-3.38E-06	5.75E-06	-0.588	0.5565
	Sex	8.75E-06	5.26E-06	1.664	0.0961
**Component**	**Term**	**edf**	**Ref. df**	**F-value**	**p-value**
Smooth Terms	s(Math Trait)	1.153	1.171	5.35E-01	0.435
	s(Math State)	1	1	1.91E+00	0.1605
	s(Time)	1	1	6.30E+00	0.0118 *
	s(Node)	8.556	8.959	1.83E+02	<2e-16 ***
	ti(Node, Math State)	5.116	6.881	1.48E+00	0.1993
	ti(Node, Time)	1	1.001	8.50E-02	0.7663

Signif. codes: 0 <= '*' < 0.001 < '**' < 0.01 < '*' < 0.05**

Adjusted R-squared: 0.778, Deviance explained 0.681

fREML: 4168.3.4, Scale est: 1.000, N: 11340

Supplemental Table 3: Summary of parametric coefficients and smooth terms for the GAMM modeling the development of mean diffusivity (MD) across the length of the left arcuate using age as the measure of time.

Component	Term	Estimate	Std. Error	t-value	p-value
Parametric Coefficients	(Intercept)	5.60E-04	1.16E-05	48.070	< 2e-16 ***
	Sex	7.49E-06	4.85E-06	1.544	0.1230

Component	Term	edf	Ref. df	F-value	p-value
Smooth Terms	s(Reading Trait)	1.001	1.001	0.71	0.39936
	s(Reading State)	1.001	1.001	2.206	0.13764
	s(Time)	1.000	1.000	5.526	0.01874 *
	s(Node)	8.841	8.994	524.236	< 2e-16 ***
	ti(Node, Reading State)	9.746	12.1	2.929	0.00052 ***
	ti(Node, Time)	8.339	11.161	2.466	0.00411 **

Signif. codes: 0 <= '*' < 0.001 < '**' < 0.01 < '*' < 0.05**

Adjusted R-squared: 0.823, Deviance explained 0.825

fREML: -221440, Scale est: 1.000, N: 19260
